# A Digital Health Fall Prevention Program for Older Adults: Feasibility Study

**DOI:** 10.2196/30558

**Published:** 2021-12-23

**Authors:** Claire L Jacobson, Lauren C Foster, Hari Arul, Amanda Rees, Randall S Stafford

**Affiliations:** 1 Age Bold Inc Los Angeles, CA United States; 2 Stanford Prevention Research Center Stanford University School of Medicine Stanford, CA United States

**Keywords:** older adults, accidental falls, fall prevention, digital health, technology, exercise, longevity and healthy aging, program evaluation, aging, elderly, health strategy

## Abstract

**Background:**

About 1 in 3 adults aged 65 and older falls annually. Exercise interventions are effective in reducing the fall risk and fall rate among older adults. In 2020, startup company Age Bold Inc. disseminated the Bold Fall Prevention Program, aiming to reduce falls among older adults through a remotely delivered, digital exercise program.

**Objective:**

We conducted a feasibility study to assess the delivery of the Bold Fall Prevention Program remotely and evaluate the program's impact on 2 primary outcomes—annualized fall rate and weekly minutes of physical activity (PA)—over 6 months of follow-up.

**Methods:**

Older adults at high risk of falling were screened and recruited for the feasibility study via nationwide digital advertising strategies. Self-reported outcomes were collected via surveys administered at the time of enrollment and after 3 and 6 months. Responses were used to calculate changes in the annualized fall rate and minutes of PA per week.

**Results:**

The remote delivery of a progressive digital fall prevention program and associated research study, including remote recruitment, enrollment, and data collection, was deemed feasible. Participants successfully engaged at home with on-demand video exercise classes, self-assessments, and online surveys. We enrolled 65 participants, of whom 48 (74%) were women, and the average participant age was 72.6 years. Of the 65 participants, 54 (83%) took at least 1 exercise class, 40 (62%) responded to at least 1 follow-up survey at either 3 or 6 months, 20 (31%) responded to both follow-up surveys, and 25 (39%) were lost to follow-up. Among all participants who completed at least 1 follow-up survey, weekly minutes of PA increased by 182% (ratio change=2.82, 95% CI 1.26-6.37, n=35) from baseline and annualized falls per year decreased by 46% (incidence rate ratio [IRR]=0.54, 95% CI 0.32-0.90, n=40). Among only 6-month survey responders (n=31, 48%), weekly minutes of PA increased by 206% (ratio change=3.06, 95% CI 1.43-6.55) from baseline to 6 months (n=30, 46%) and the annualized fall rate decreased by 28% (IRR=0.72, 95% CI 0.42-1.23) from baseline to 6 months.

**Conclusions:**

The Bold Fall Prevention Program provides a feasible strategy to increase PA and reduce the burden of falls among older adults.

## Introduction

Every year, one-third of community-dwelling older adults (adults aged 65 and older) experience a fall [1]. Falls, defined as “unexpected event[s] in which the participant comes to rest on the ground, floor, or lower level,” are responsible for a wide range of negative health outcomes [2]. Annually, more than 3 million older Americans are treated in emergency departments for fall injuries, and over 800,000 are hospitalized, often because of a head injury or hip, spine, or wrist fracture [3]. Falls are the leading cause of injury-related deaths among older adults, and the age-adjusted fall death rate (64 deaths per 100,000 older adults) increased by 30% from 2009 to 2018 [4]. Additionally, the psychological impact of falling can cause older adults and their caregivers significant fear about the risk of falling again. This fear of falling can have an accumulating effect whereby the fear of falling causes individuals to limit their everyday physical activities, which in turn makes them weaker and more susceptible to future falls. In fact, studies have shown that falling once doubles the chances of falling again [5].

These negative physical and psychological impacts of falling are accompanied by a staggering price tag for the American health care system. According to the Centers for Disease Control and Prevention (CDC), the direct medical costs for falls in 2015 alone totaled more than $50 billion, and 75% of those costs were shouldered by Medicare and Medicaid [6]. On average, the direct medical cost of hospitalization for a fall-related injury is $30,000 and increases with age [7]. As the number of older adults in the United States continues to grow, so too will the projected fall-related costs.

Many falls, however, can be prevented. One of the most effective ways to reduce fall risk is through targeted exercise that improves an individual’s strength, balance, and mobility [8]. Exercise-based programs, such as the Otago Exercise Program and Tai Ji Quan: Moving for Better Balance, have been shown to reduce falls by up to 35% and 55%, respectively [9,10]. Until recently, however, the vast majority of fall prevention programs were only offered in small, in-person classes hosted in local senior centers or gyms. Although this has been the standard dissemination method for decades, it comes with significant barriers to participation. Common barriers to in-person programs include a lack of programs in rural or underresourced communities; limited or no access to transportation; scheduling conflicts; cost of getting to and using facilities; interpersonal barriers, such as finding other participants’ presence intimidating; and physical environmental barriers, such as bad weather, stairs, uneven ground, difficult parking, and more [11].

In the past decade, trends in the United States indicate substantial movement toward the use of digital health tools for older adults. Between 2013 and 2017, smartphone ownership by those aged 65 and older more than doubled from 18% to 42% [12]. Similarly, 67% of all US older adults report that they spend time online [12]. Leveraging technology to facilitate remote fall prevention exercise programs provides an exciting opportunity to make a meaningful impact in elder care by increasing access to training and resources and removing barriers that have limited the reach of previous efforts. To meet this growing need, Age Bold Inc. (Bold), a San Francisco–based digital health company, created the Bold Fall Prevention Program. Here, we describe the results and impacts of the program’s feasibility study launched in January 2020, including an evaluation of the two primary outcomes—annualized fall rates and minutes of physical activity (PA)—at 6-month follow-up.

## Methods

### Purpose and Objectives

The feasibility study for the Bold Fall Prevention Program was designed to demonstrate the ability to remotely deliver an innovative digital exercise program. We assessed the program’s ability to engage and retain participants and to remotely collect outcomes data. The study also examined the program’s impact on fall rates and measures of physical function in older adults over 6 months, with continued follow-up to 12 months. The research study was approved by the WIRB-Copernicus Group® (Puyallup, WA, USA) institutional review board (#520190289).

### Intervention Approach

Bold’s Fall Prevention Program is a 12-week digital program of progressive exercise routines aimed at increasing strength, mobility, and balance to reduce the risk of falls. All exercise sessions (classes) were provided online and on demand. Every session was guided by Bold instructors, which included kinesiologists, personal trainers, and community Tai Chi instructors. The program included exercises common to evidence-based programs, such as Stay Active and Independent for Life (SAIL), FallProof, Matter of Balance, and Tai Ji Quan: Moving for Better Balance.

Bold’s classes were designed to progressively build physical strength, balance, and mobility and included static and dynamic movements that challenged a participant’s center of gravity (eg, tandem walking). The addition of weight-shifting and overreaching exercises progressively increased the challenge of these exercises throughout the duration of the program. The classes included a variety of exercises for activation of the anterior and posterior of the lower leg and foot muscles (eg, plantar and dorsiflexion), for hip activation (eg, squats, lunges, lateral leg lifts, knee lifts, and hip extensions), and for postural control (eg, chest stretches, chin tucks, and rows). Hamstring curls, hip extensions, hip flexion, and leg extensions were incorporated to build lower body strength, specifically in the quadriceps, glutes, and hamstrings. Side bends, knee to elbow, trunk rotations, and static abdominal holds were added to build upper and lower back strength. The program classes also included instruction of 8 Tai Chi forms in seated or standing positions (eg, parting wild horse's mane, single whip, wave hands like clouds, repulse monkey, brush knee, grasp the peacock's tail, and fair lady works shuttles). The majority of Bold programming combined elements from evidence-based fall prevention programs, and additional exercises were designed and taught by a kinesiologist with more than a decade of experience working with older adults.

Class durations ranged from 30 to 45 minutes per class, 3 times a week, for a minimum of 100 minutes of class instruction per week. This exercise dosage was based on findings from early pilot testing and is in conjunction with other validated fall prevention programs [13,14]. Participants were encouraged to take a Bold class at least 2 times per week, supplemented by other light PA, like walking, on most other days.

Based on the participants’ stated preferences and abilities, collected at baseline, they received a customized program that began with either seated or standing exercises and progressed in difficulty over time. Ankle weights and a balance prop (a balance disc for participants beginning seated and a balance pad for participants beginning standing) were shipped to participants upon enrollment in the program. In the seated exercise progression, ankle weights were introduced for strength-focused sessions, a balance disc was used to build core strength and postural control in advanced seated balance exercises, and as the program progressed, participants gradually began incorporating standing exercises into their routines. In the standing progression, ankle weights were introduced for strength-focused sessions, and a foam balance pad was introduced for advanced standing balance exercises.

A typical class structure included a dynamic warm-up; a balance, strength, or combination balance-and-strength segment; and a cool-down (Multimedia Appendix 1). In each class, instructors provided safety guidelines and instructions for when to use props or modify various exercises. Participants were also encouraged to reach out to the instructors and research team at the end of each class with questions about using the props and to get further guidance, if so desired.

In addition to weekly exercise classes, participants received educational and motivational support, including weekly emails with resources to learn more about balance and fall prevention, individual motivational text messages, and phone calls. After the initial 12-week program was completed, participants progressed to a 9-month “maintenance” phase with access to additional exercise classes to maintain their progress.

### Recruitment

Potential participants were identified and recruited through nationwide digital strategies and physical flyers placed in local communities in California. Prospective participants were directed to learn more about the study on the study website.

Eligible participants were 65 years old or older, had reliable computer or tablet access with broadband internet, and were able to consent and follow study instructions in English. In addition, they met 1 or more of the following criteria: (1) a history of 1 or more injurious fall(s) in the past 12 months, (2) a history of 2 or more non-injurious falls in the past 12 months, (3) reduced mobility indicated by a Timed Up & Go test score of ≥12 seconds (“mobility assessment”), (4) reduced balance indicated by an inability to hold positions 3 or 4 for ≥10 seconds on the 4-Stage Balance Test (“balance assessment”), or (5) reduced strength indicated by a score below average for age/gender on the 30-second Chair Stand Test (“strength assessment”).

The mobility, balance, and strength assessments were directly adapted from the CDC’s Stopping Elderly Accidents, Deaths, and Injuries functional assessments [15]. These assessments were adapted to the Bold platform through several rounds of user testing prior to the start of the study. During these sessions, older adult participants would conduct the self-assessment while a member of the research team observed. Feedback gathered from participants and the research team during these sessions informed how the self-assessments were presented on the digital platform and the guidance provided to study participants. Delivered remotely via the Bold website during the eligibility screening, participants watched an instructional video in which a Bold instructor explained how to complete the self-assessment and demonstrated each step in the process. Participants were able to watch the video as often as they wanted before they began the assessment. For the strength and mobility self-assessments, participants were provided with a digital stopwatch that they could control, as needed. For the strength assessment, they were instructed to count the number of repetitions they could complete within the allotted time. For the mobility assessment, they could start and stop the stopwatch, as needed, and adjust their final time to accommodate any delays between completing the assessment and hitting the digital stopwatch.

Participants were excluded if they met any of the following criteria: (1) regular use of a mobility aid, such as a walker, wheelchair, or scooter; (2) any contraindication to light PA; (3) neurological impairments that impact gait (eg, Parkinson’s disease); (4) cognitive impairment above a level consistent with safety; (5) severely reduced measures of physical function (eg, unable to complete functional assessments); and (6) at the clinical discretion of investigators.

In total, 65 participants who met the eligibility requirements were enrolled in the study. Each participant received $10 for completion of the baseline survey, $10 for completion of the 3-month survey, and $20 for completion of the 6-month survey.

### Data Collection and Statistical Analysis

Eligibility and outcome data were remotely collected via questionnaires on Bold’s website during the enrollment process. Follow-up surveys were administered via Typeform, an online survey tool that allows the remote delivery, collection, and tracking of study surveys to participants.

To evaluate the feasibility of the intervention, we focused on 3 areas: (1) the feasibility of remote enrollment of a population at high risk of falls, (2) the feasibility of remote data collection, and (3) the feasibility of digitally delivering a progressive fall prevention exercise program.

To assess the program’s impact on PA, participants were asked to think about the previous 7 days and report how many minutes of PA (including walking) they did each day. Responses were used to estimate the weekly minutes of PA at baseline, 3 months, 6 months, and a combined 3-and 6-month estimate (weighted by the person-years since the last survey). Weekly minutes of PA were logarithmically transformed to normalize the data and allow for statistical analysis. Unpaired and paired 2-tailed *t* tests were used to calculate mean group differences of the log-transformed minutes of PA. Exponentiating these differences provided estimates of the ratio change from baseline.

To evaluate the program’s impact on fall rates and weekly minutes of PA, enrolled participants completed surveys at the time of enrollment (baseline survey), 3 months after enrollment, and 6 months after enrollment. In each survey, participants were asked to report the total number of falls experienced, if any, since the last survey. At 3 and 6 months, the annualized fall rate was calculated as the total number of falls reported divided by 0.25 and 0.5 person-years, respectively [1,14,16]. For participants who completed both surveys, the total number of falls at 6 months was inclusive of any falls previously reported in the 3-month survey. Changes in annualized falls rates were calculated as incidence rate ratios (IRRs) from baseline to 3 months, 6 months, and combined 3 and 6 months. All change estimates are reported with 95% CIs and 2-tailed *P*-values. Descriptive statistical methods, including mean, standard deviations, and frequencies, were also used to summarize study data. Statistical analyses were performed using R Studio software (Boston, MA, USA).

## Results

### Demographic Characteristics of Participants

The demographic characteristics of the study population are presented below (Table 1). The study population included 65 participants: 48 (74%) women and 17 (26%) men. Of these, 60 (92%) participants identified as White, and the mean age was 72.6 years. The highest education level attained was high school or equivalent for 7 (11%) participants, some college or associate degree for 16 (25%) participants, and college graduate or higher for 42 (65%) participants. In addition, 58 (89%) participants had Medicare or Medicare Advantage as their health insurance. Furthermore, 30 (46%) participants had at least 1 chronic condition and 12 (19%) had at least 5 prescribed medications. The majority (39/65, 60%) of participants had fallen at least 1 time in the previous 12 months, and on average, participants engaged in 151 minutes of PA per week, although almost half (30/65, 46%) of the participants reported engaging in less than 1 hour of PA per week.

**Table 1:** Baseline characteristics of study participants (N=65).

Characteristics		Number of participants, n (%)
**Gender**		
	Female	48 (74)
	Male	17 (26)
**Age in years**		
	65-69	25 (39)
	70-74	22 (34)
	75-79	8 (12)
	80 and older	10 (15)
**Highest education level**		
	High school diploma or equivalent	7 (10)
	Some college or associate degree	16 (25)
	College graduate or higher	42 (65)
**Race/ethnicity**		
	White	60 (93)
	American Indian or Alaska Native	1 (1)
	Asian (including South Asian and Asian Indian)	1 (1)
	Black or African American	2 (4)
	Prefer not to state	1 (1)
**Income in US $**		
	<20,000	9 (14)
	20,000-49,999	21 (32)
	50,000-74,999	18 (28)
	75,000-99,999	8 (12)
	≥100,000	5 (8)
	Prefer not to state	4 (6)
**Geographic region**		
	West	20 (30)
	Midwest	14 (21)
	Northeast	17 (26)
	South	14 (22)
**Insurance**		
	Medicare	40 (61)
	Medicare Advantage Private Plan (Medicare Part C)	18 (28)
	Employer-based insurance	5 (8)
	Veterans Affairs Health Care	2 (3)
**Chronic medical conditions^a^**		
	Any chronic medical condition	30 (46)
	Cardiovascular disease	9 (14)
	Type 2 diabetes	12 (19)
	Musculoskeletal condition	24 (37)
	Chronic obstructive pulmonary disorder (COPD)/other lung disease	5 (8)
	Depression	7 (11)
	Other	12 (19)
**Number of prescribed medications**		
	None	15 (23)
	1-4	38 (59)
	≥5	12 (18)
**Fall in previous 12 months**		
	Yes	39 (60)
	No	26 (40)
**Amount of weekly PA^b^ in minutes**		
	<60	29 (45)
	61-120	9 (14)
	121-180	9 (14)
	181-240	4 (6)
	241-300	4 (6)
	201-360	1 (1)
	>360	7 (11)

^a^Multiple responses were possible.

^b^PA: physical activity.

### Feasibility

In all, 143 high-fall-risk older adults were recruited between January and February 2020. Tracking showed that 100% of the eventually enrolled participants were found via digital ads on Facebook. Of the 143 older adults recruited, 65 (45.5%) completed enrollment. Most of this drop-off can be attributed to individuals who began the enrollment process because they were curious, but ceased involvement after learning about the time commitment required for participation. The process of remote enrollment via online surveys and self-assessments was successful in identifying and engaging high-fall-risk older adults in the study. Participants did not report any issues with completing the surveys or administering the self-assessments.

Of the 65 enrolled participants, 54 (83%) took at least 1 class during the study period, 30 (46%) took at least 10 classes, and 14 (22) took at least 35 classes. The average number of classes taken was 18 (SD 21, median 7, 25th percentile=1, 75th percentile=33). In addition, 29 (45%) and 31 (48%) participants completed the 3- and 6-month surveys, respectively, indicating that the remote data collection process was only moderately successful in achieving high levels of survey completion. Operationally, automated data collection, tracking, and alert notifications were beneficial to the research team and greatly diminished the need for a large research staff. However, during the collection of the 3-month follow-up survey, the research team noticed that most participants required between 1 and 3 phone calls to prompt them to complete their survey, suggesting that establishing a connection with a member of the research team was an integral part of the data collection process. Accordingly, it was determined that although the process of remote data collection provides distinct benefits for a research team working with a geographically distributed population, this modality requires additional support to mitigate substantial losses to follow-up. These findings indicate that it is feasible for older adults to adhere to a digital exercise program; however, they would likely benefit from supplemental outreach to encourage consistent class-taking and engagement behavior.

From the perspective of both the participants and the research team, the digital delivery of a progressive fall prevention exercise program was feasible. Participants were able to engage in the program and follow along week by week, even during the COVID-19 pandemic. Many participants reported the program as their sole form of exercise due to the many of the pandemic-related restrictions put in place, and stated that they wished to continue exercising at home even after the restrictions were lifted. This suggests that the digital exercise program addresses a previously unmet need in the community and its delivery was sufficient to engage those community members. For the research team, in addition to strong relationships with the engineering team, which allowed for quick troubleshooting of technical issues, traditional methods, such as calling and emailing participants, to check in and establish connections were crucial. Doing so allowed the research team to answer participant questions quickly, anticipate future challenges, and proactively respond to aspects of the program experience that may be confusing. Overall, the remote delivery of the program was sufficient to establish a basis for the further development and expansion of the digital fall prevention exercise program.

### Physical Activity

The change in weekly minutes of physical activity for all participants in shown in Table 2. Paired comparisons showed that weekly minutes of PA were 3.06 times higher (95% CI 1.43-6.55) at 6 months than at baseline (n=30, 46%; Table 3). At 3 months (n=18, 28%), weekly minutes of PA were 1.49 times higher (95% CI 0.60-3.72) than at baseline (Table 3). The combined estimate of all participants who submitted at least 1 follow-up survey with completed PA data (n=35, 54%) showed that weekly minutes of PA were 2.82 times higher (95% CI 1.26-6.37) than at baseline (n=35, 54%), a 182% increase (Table 3).

**Table 2:** Change in weekly minutes of physical activity for all participants (N=65).

Time of survey, n (%)	Mean PA^a^ in minutes	Mean log PA in minutes	Log SD	Difference from baseline in log minutes, n (95% CI)	Ratio change in minutes, n (95% CI)	*P* value^b^
Baseline, 63 (97)	151	1.86	0.61	Reference group	Reference group	—^c^
3 months, 18 (28)	251	2.24	0.58	0.38 (0.055-0.69)	2.37 (1.14-4.90)	.02
6 months, 30 (46)	456	2.38	0.65	0.52 (0.23-0.80)	3.31 (1.71-6.31)	<.001
Combined responders, 35 (54)	396	2.34	0.78	0.48 (0.17-0.78)	3.02 (1.49-6.07)	.003

^a^PA: physical activity.

^b^*P* value from *t* test.

^c^Not applicable.

**Table 3:** Subcohort analysis: baseline weekly minutes of physical activity by survey response group (N=65).

Time of survey, n (%)	Mean PA^a^ in minutes	Mean log PA in minutes	Log SD	Difference from baseline in log minutes, n (95% CI)	Ratio change in minutes, n (95% CI)	*P* value^b^
Baseline: 3-month responders, 18 (28)	192	2.06	0.57	0.17 (–0.22 to 0.57)^c^	1.49 (0.60-3.72)^c^	.37
Baseline: 6-month responders, 30 (46)	175	1.89	0.67	0.49 (0.16-0.82)^d^	3.06 (1.43-6.55)^d^	.01
Baseline: combined responders, 35 (54)	162	1.89	0.63	0.45 (0.10-0.80)^e^	2.82 (1.26-6.37)^e^	.01

^a^PA: physical activity.

^b^*P* value from *t* test.

^c^Calculated as 3 months vs baseline: 3-month responders.

^d^Calculated as 6 months vs baseline: 6-month responders.

^e^Calculated as combined responders vs baseline: combined responders.

### Falls

Of the 65 enrolled participants, 40 (62%) responded to at least 1 follow-up survey at either 3 or 6 months, 20 (31%) responded to both follow-up surveys, and 25 (39%) were lost to any follow-up. The change in the annualized fall rate for all participants is shown in Table 4. Among 6-month survey responders (n=31, 48%), the annualized fall rate decreased by 28% (IRR=0.72, 95% CI 0.42-1.23) from baseline to 6 months (Table 5). Among 3-month survey responders (n=29, 45%), the annualized fall rate per person decreased by 83% (IRR=0.17, 95% CI 0.05-0.55) from baseline to 3 months (Table 5). The combined estimate of all participants who completed at least 1 follow-up survey (n=40, 62%) showed a 46% reduction in the annualized fall rate (IRR=0.54, 95% CI 0.32-0.90) from baseline (Table 5).

**Table 4:** Change in annualized fall rate for all participants (N=65).

Time of survey, n (%)	Fall rate (falls per person-year), n	SD	IRR^a^ (95% CI)	*P* value^b^
Baseline, 65 (100)	1.42	1.98	Reference group	—^c^
3 months, 29 (45)	0.41	1.64	0.29 (0.09-0.92)	.03
6 months, 31 (48)	1.16	3.09	0.82 (0.50-1.36)	.44
Combined responders, 40 (62)	1.01	1.17	0.72 (0.43-1.19)	.19

^a^IRR: incidence rate ratio.

^b^*P* value from the IRR.

^c^Not applicable.

**Table 5:** Subcohort analysis: baseline fall rate by survey response group (N=65).

Time of survey, n (%)	Fall rate (falls per person-year), n	SD	IRR^a^ (95% CI)	*P* value^b^
Baseline: 3-month responders, 29 (45)	2.38	2.53	0.17 (0.05-0.55)^c^	<.001
Baseline: 6-month responders, 31 (48)	1.61	2.16	0.72 (0.42-1.23)^d^	.23
Baseline: combined responders, 40 (62)	1.88	2.32	0.54 (0.32-0.90)^e^	.02

^a^IRR: incidence rate ratio.

^b^*P* value from the IRR.

^c^Calculated as 3 months vs baseline: 3-month responders.

^d^Calculated as 6 months vs baseline: 6-month responders.

^e^Calculated as combined responders vs baseline: combined responders.

**Figure 1:**
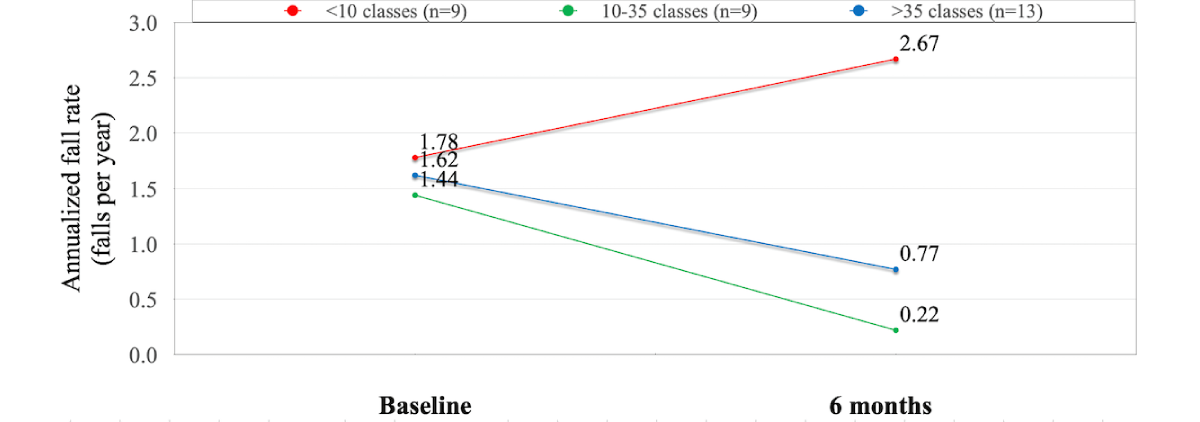
Change in the annualized fall rate per person between baseline and 6 months, stratified by class-taking behavior, for 31 of 65 participants (48%) who completed their 6-month survey.

The change in the annualized fall rate correlated with the number of Bold classes taken among the 6-month survey responders. Participants who took fewer than 10 classes (n=9, 29%) reported an increase in the annualized fall rate from 1.78 annualized falls per person at baseline to 2.67 at 6 months, while those who took 10-35 classes (n=9, 29%) showed a decreased fall rate from 1.44 at baseline to 0.22 at 6 months, and those who took more than 35 classes (n=13, 42%) also showed a decrease from 1.62 at baseline to 0.77 at 6 months (Figure 1).

There were some differences in baseline demographics between the groups. Of those who took more than 35 classes (n=13, 42%), 4 (31%) were over the age of 80 as compared to 1 (11%) in each of the other 2 cohorts (n=9 each). Of those who took fewer than 10 classes (n=9, 29%), 5 (56%) had experienced 1 or more falls in the 12 months prior to joining the study compared to 6 (67%) and 8 (62%) of those who took 10-35 (n=9, 29%) and more than 35 (n=13, 42%) classes, respectively. This cohort also had lower levels of average baseline PA as compared to those who took 10–35 or more than 35 classes, engaging in 116 minutes per week compared to 191 and 207 minutes, respectively. Further comparisons of demographic characteristics by class-taking behavior can be found in Multimedia Appendix 2.

Significant loss to follow-up was observed at 3 and 6 months. Baseline fall rates and PA levels, however, were similar for 3- and 6-month responders compared to all participants. This suggests that follow-up was not biased toward participants with higher baseline levels of PA.

## Discussion

### Principal Findings

The feasibility study findings indicate that it is possible to digitally disseminate this exercise-based fall prevention intervention and remotely study its impact on outcomes. At present, the majority of fall prevention programs occur in-person at local community centers, hospitals, or gyms, so the potential to deliver customized, digital-first programming could significantly increase the accessibility of such programs to people who cannot use the in-person offerings. It is also significant to the continued development of future studies of this intervention and provides useful guidance for future study design. From an effectiveness perspective, the study suggests that the Bold Fall Prevention Program has a positive impact on fall- and PA-related outcomes that endures even after the initial 3-month intervention window, with a notable 206% increase in PA from baseline to 6 months. The remote implementation and evaluation of a digital fall prevention program among older adults has the potential for significant health and cost impact among the rapidly growing older adult population in the United States.

### Comparison With Prior Work

Compared with in-person interventions, the Bold program reduced barriers to participant engagement by eliminating transportation challenges, allowing for self-scheduling of classes, customizing class content to match individuals’ needs, and removing the need to have a local program facilitator or fitness center. An entirely digital program has the added benefits of scalability and lower distribution costs compared with traditional fall prevention programs.

Notably, the feasibility study was initiated just before the COVID-19 pandemic emerged in early 2020. Hence, participants likely were impacted by the countrywide trends of increased time spent at home, decreased PA, and increased feelings of isolation and stress, particularly for older adults who are most at risk for developing severe COVID-19 symptoms [17]. At the same time, COVID-19 provided a unique opportunity to disseminate and evaluate a digital health tool. The reliance on and growth of the remote delivery of health care and exercise will likely persist in the postpandemic world [18].

Among participants who responded to at least 1 follow-up survey (n=40, 62%), the statistically significant 46% reduction in the annualized fall rate (IRR=0.54) is slightly greater than that reported by the US Preventive Services Task Force (USPSTF) synthesis. The USPSTF data synthesis indicated that multifactorial PA interventions based on initial fall risk assessments are, on average, associated with a 21% reduction in the fall rate (IRR=0.79, 95% CI 0.68-0.91) [19]. Additionally, the observed 46% reduction in the fall rate during follow-up is comparable to the fall reductions reported by the Otago Exercise Program (reduced falls by 35%) and Tai Ji Quan: Moving for Better Balance (reduced falls by 55%), which are 2 of the most widely disseminated fall prevention programs in the United States [9,10].

### Limitations

There were several limitations of the feasibility study, including the number of participants lost to follow-up and the resultantly small sample size, reliance on self-reported measures for falls and PA, inconsistent survey response rates, and the lack of a control population, instead relying on before-after assessment of outcomes. It is hypothesized that the high loss to follow-up seen in the follow-up surveys was the result of a mix of contributing factors, including (1) user experience of difficulty when participants were directed to a website external to the Bold web application for the completion of their follow-up surveys, (2) a loss of the sense of personal connection and contribution to science as a result of the fully remote and digital nature of the study and study setting, and (3) the natural attrition that occurs in a study population over time. This is particularly informative as a lack of engagement and loss to follow-up are a particular concern for remote studies. Although the data presented in this paper are preliminary and best suited to be used as an early foundation for more formal investigations, the findings about the role that interpersonal connection, even over the phone or via email, can play in mitigating this loss to follow-up are important and should be strongly considered when planning future studies of this intervention.

The feasibility study was designed to demonstrate the ability to remotely deliver an innovative digital exercise program and provide preliminary evidence about the effectiveness of that program while also not obstructing the fast-paced and iterative nature of a startup. As such, Bold decided to conduct this feasibility study rather than immediately launching a resource-intensive and expensive randomized controlled trial. Although the research pursued is still valuable and unique within the for-profit digital health landscape, the findings are subject to more bias, confounding, and other systematic errors than would those of a randomized controlled trial.

The study did not define a clear minimum number of Bold classes that participants needed to complete to be included in the analysis. It is thought that 29 classes constitute a “full dose” of programming based on the number of minutes of strength, balance, and mobility training delivered per week, as well as the total number of targeted training sessions someone would typically receive after 29 classes (~18 hours). Although this is in line with other programs, such as SAIL and Matter of Balance, further research and investigation into this threshold is warranted. Additionally, we considered that enrolling into the program demonstrated an intention to reduce one’s risk of falling and thus is an important early indicator of a subsequent reduction in falls. Accordingly, further investigation is warranted into the effect of class-taking behavior on the effectiveness of the intervention. These limitations notwithstanding, this feasibility study is valuable for assessing the basic feasibility and practicality of the intervention as well as for identifying areas for product improvements and modifications.

### Future Directions

Bold has used these study findings and participants’ feedback to improve its current, publicly available product. Based on this feasibility study, we plan to conduct a clinical trial that rigorously assesses the impact of the program on fall rates, physical function, and health care costs in a Medicare Advantage population. Bold as a company is growing and widening its aperture to expand its video and nonvideo content to support more lasting behavior change and provide a more comprehensive toolkit to reduce older adults’ likelihood of falling and improve their healthspan.

### Conclusion

Falls pose a significant public health threat for the increasing older adult population. Multifactorial and exercise-based fall prevention programs can reduce the risk of falls, yet the current landscape of fall prevention programs largely consists of fragmented, in-person, resource-intensive programs. The movement toward digital health tools, accelerated by the COVID-19 pandemic, highlights the timeliness and potential value of a digital exercise program designed specifically for older adults [12,15]. The Bold Fall Prevention Program leverages technology to provide an individually tailored program of online and on-demand exercise classes aimed at preventing falls. Preliminary results from this feasibility study suggest that the Bold Fall Prevention Program, with its potential for expanding access, could be a useful tool in reducing falls and increasing PA in older adults.

## Appendix

Multimedia Appendix 1 Exercise program progressions and sample class plans.

Multimedia Appendix 2 Participant demographics by class-taking behavior.

## Abbreviations

CDC: Centers for Disease Control and Prevention
IRR: incidence rate ratio
PA: physical activity
SAIL: Stay Active and Independent for Life
USPSTF: US Preventive Services Task Force
