# Supplementary material for: A Digital Health Fall Prevention Program for Older Adults: Feasibility Study
Source: JMIR Form Res. 2021 Dec 23;5(12):e30558. doi: 10.2196/30558 (PMC8738986; doi:10.2196/30558)
Supplement: Multimedia Appendix 1 [file formative_v5i12e30558_app1.docx]

| **Progression of exercise challenge over 3 months** | | | |
| --- | --- | --- | --- |
|  | Month 1 | Month 2 | Month 3 |
| Standing Program | Exercises without weights, option to use chair for balance support | Incorporate ankle weights and multitasking for dynamic balance | Introduce balance pad in some exercises to challenge stability on uneven surfaces |
| Seated Program | Begin with bodyweight strength exercises and incorporate ankle weights at week 3. | Incorporate balance disc to challenge postural stability and press to some standing exercises | Increase duration of standing exercises |

| **Sample Standing Balance Class (Week 2)** | |
| --- | --- |
| Introduction | Introduction & Safety Guidance |
| Warm Up | Deep breaths, pelvic tilts, gentle twists, shoulder rolls, head turns, neck tucks, form a ball, ankle circles, wind ups |
| Balance | Moving a Ball (Tai Chi),  Part the Wild Horse’s Mane (Tai Chi), Closing Form (Tai Chi) |
| Cool Down | Neck tilts, head rolls, side lunges, forward lunge, hip circles, wrist stretches, back extension, deep breaths |

| **Sample Seated Class (Week 2)** | |
| --- | --- |
| Introduction | Introduction & Safety Guidance |
| Warm Up | Ankle circles, cat/cow, neck tucks, head turns, elbow circles, gentle twists, back extension, seated march, side leg lifts, seated knee extensions, marching with head turns, heel-toe steps, shoulder shrugs |
| Balance | Hip rocks (challenge posture), seated weight shifts forwards and backwards, weight shifts with arm reaches side to side, seated crunches |
| Strength | Sit to stand, seated hamstring curls, knee extensions, glute squeeze, heel raises |
| Cool Down | Wrist stretch, forward folds, side neck stretch, back extension, quadricep stretch, hamstring stretch, inner thigh stretch, head turns, arm circles, write circles, hand/ finger stretch, back scratch |
