# Supplementary material for: A Digital Health Fall Prevention Program for Older Adults: Feasibility Study
Source: JMIR Form Res. 2021 Dec 23;5(12):e30558. doi: 10.2196/30558 (PMC8738986; doi:10.2196/30558)
Supplement: Multimedia Appendix 2 [file formative_v5i12e30558_app2.docx]

| **Characteristics** | | **<10 Classes  (N=9)** | **10 - 35 Classes**  **(N=9)** | **35+ Classes**  **(N=13)** |
| --- | --- | --- | --- | --- |
| **Gender, n (%)** | |  |  |  |
|  | **Female** | 7 (77.8) | 7 (77.8) | 9 (69.2) |
|  | **Male** | 2 (22.8) | 2 (22.8) | 4 (30.8) |
| **Age, n (%)** | |  |  |  |
|  | **65-69** | 4 (44.4) | 3 (33.3) | 7 (53.8) |
|  | **70-74** | 1 (11.1) | 5 (55.6) | 2 (15.4) |
|  | **75-79** | 3 (33.3) | 0 (0.0) | 0 (0.0) |
|  | **80 and older** | 1 (11.1) | 1 (11.1) | 4 (30.8) |
| **Highest Education Level, n (%)** | |  |  |  |
|  | **High school diploma or equivalent** | 1 (11.1) | 0 (0.0) | 1 (7.7) |
|  | **Some college or associate degree** | 3 (33.3) | 1 (11.1) | 3 (23.1) |
|  | **College graduate or higher** | 5 (55.6) | 8 (88.9) | 9 (69.2) |
| **Race/Ethnicity, n (%)** | |  |  |  |
|  | **White** | 9 (100) | 8 (88.9) | 13 (100) |
|  | **American Indian or Alaska Native** | 0 (0.0) | 0 (0.0) | 0 (0.0) |
|  | **Asian (including South Asian and Asian Indian)** | 0 (0.0) | 0 (0.0) | 0 (0.0) |
|  | **Black or African-American** | 0 (0.0) | 1 (11.1) | 0 (0.0) |
|  | **Prefer not to state** | 0 (0.0) | 0 (0.0) | 0 (0.0) |
| **Income, n (%)** | |  |  |  |
|  | **<$20,000** | 2 (22.2) | 0 (0.0) | 1 (7.7) |
|  | **$20,000-49,999** | 4 (44.4) | 1 (11.1) | 6 (46.2) |
|  | **$50,000-74,999** | 1 (11.1) | 6 (66.7) | 4 (30.8) |
|  | **$75,000-99,999** | 0 (0.0) | 1 (11.1) | 1 (7.7) |
|  | **$100,000+** | 2 (22.2) | 1 (11.1) | 1 (7.7) |
|  | **Prefer not to state** | 0 (0.0) | 0 (0.0) | 0 (0.0) |
| **Geographic Region, n (%)** | |  |  |  |
|  | **West** | 1 (11.1) | 3 (33.3) | 3 (23.1) |
|  | **Midwest** | 1 (11.1) | 3 (33.3) | 3 (23.1) |
|  | **Northeast** | 3 (33.3) | 1 (11.1) | 3 (23.1) |
|  | **South** | 4 (44.4) | 2 (22.2) | 4 (30.8) |
| **Insurance, n (%)** | |  |  |  |
|  | **Medicare** | 5 (55.6) | 3 (33.3) | 7 (53.8) |
|  | **Medicare Advantage Private Plan (Medicare Part C)** | 4 (44.4) | 3 (33.3) | 4 (30.8) |
|  | **Employer-based insurance** | 0 (0.0) | 3 (33.3) | 1 (7.7) |
|  | **Veterans Affairs Health Care** | 0 (0.0) | 0 (0.0) | 1 (7.7) |
| **Chronic Medical Conditions, n (%)** | |  |  |  |
|  | **Any Chronic Medical Condition** | 5 (55.6) | 6 (66.7) | 7 (53.8) |
|  | **Cardiovascular Disease** | 4 (44.4) | 0 (0.0) | 1 (7.7) |
|  | **Type 2 Diabetes** | 3 (33.3) | 2 (22.2) | 1 (7.7) |
|  | **Musculoskeletal Condition** | 3 (33.3) | 5 (55.6) | 3 (23.1) |
|  | **COPD / Other Lung Disease** | 2 (22.2) | 0 (0.0) | 0 (0.0) |
|  | **Depression** | 1 (11.1) | 2 (22.2) | 0 (0.0) |
|  | **Other** | 0 (0.0) | 2 (22.2) | 4 (30.8) |
| **Number of prescribed medications, n (%)** | |  |  |  |
|  | **None** | 0 (0.0) | 2 (22.2) | 3 (23.1) |
|  | **1 to 4** | 1 (11.1) | 6 (66.7) | 9 (69.2) |
|  | **5 or more** | 8 (88.9) | 1 (11.1) | 1 (7.7) |
| **Fall in previous 12 months, n (%)** | |  |  |  |
|  | **Yes** | 5 (55.6) | 6 (66.7) | 8 (61.5) |
|  | **No** | 4 (44.4) | 3 (33.3) | 5 (38.5) |
| **Amount of weekly physical activity, n (%)** | |  |  |  |
|  | **Less than 60 minutes** | 4 (44.4) | 3 (33.3) | 3 (25.0) |
|  | **61 to 120** | 2 (22.2) | 2 (22.2) | 3 (25.0) |
|  | 121 to 180 | 1 (11.1) | 1 (11.1) | 0 (0.0) |
|  | **181 to 240** | 0 (0.0) | 1 (11.1) | 2 (16.7) |
|  | **241 to 300** | 1 (11.1) | 1 (11.1) | 1 (8.3) |
|  | **201 to 360** | 0 (0.0) | 0 (0.0) | 1 (8.3) |
|  | **More than 360 minutes** | 1 (11.1) | 1 (11.1) | 2 (16.7) |
